# Interventions in ambulatory healthcare settings to reduce social isolation among adults aged 18–64: a systematic review

**DOI:** 10.3399/BJGPO.2023.0119

**Published:** 2024-11-13

**Authors:** Kavya Anchuri, Liane Steiner, Roxana Rabet, Amy Craig-Neil, Ellah San Antonio, Oluwasegun Jko Ogundele, Melanie Seabrook, Ceinwen Pope, Serina Dai, Andree Schuler, Carolyn Ziegler, Andrew David Pinto

**Affiliations:** 1 Upstream Lab, MAP Centre for Urban Health Solutions, Li Ka Shing Knowledge Institute, Unity Health Toronto, Toronto, Canada; 2 Dalla Lana School of Public Health, University of Toronto, Toronto, Canada; 3 Department of Family and Community Medicine, St. Michael’s Hospital, Toronto, Canada; 4 Institute for Health Policy, Management and Evaluation, Dalla Lana School of Public Health, University of Toronto, Toronto, Canada; 5 Library Services, Unity Health Toronto, St. Michael’s Hospital, Toronto, Canada; 6 Department of Family and Community Medicine, Faculty of Medicine, University of Toronto, Toronto, Canada

**Keywords:** social isolation, loneliness, primary care

## Abstract

**Background:**

Social isolation is associated with increased all-cause and premature mortality, poor chronic disease management, and mental health concerns. Limited research exists on interventions addressing social isolation among individuals under 65 despite its increasing prevalence among young and middle-aged adults.

**Aim:**

To identify interventions from the extant literature that address social isolation and loneliness in ambulatory healthcare settings in adults aged 18–64 and to identify elements of successful studies for future intervention design.

**Design & setting:**

Systematic review of interventions targeting social isolation in community-dwelling adults aged 18–64 within ambulatory healthcare settings.

**Method:**

A search strategy was developed to identify relevant articles in the following databases: Ovid MEDLINE, Embase, EBM Reviews, Scopus, CINAHL, and PsychInfo. Data were extracted on study design and setting, intervention type, outcome related to social isolation/loneliness, and scale of measure used.

**Results:**

25 078 citations were identified and underwent title and abstract screening. 75 articles met our inclusion criteria and were synthesised, including an assessment of bias. Effective interventions were delivered in community health settings, incorporated a group component, and used digital technologies. They also addressed the association between mental health and social isolation using cognitive-behavioural therapy (CBT) approaches and enhanced self-management and coping strategies for chronic conditions through psycho-educational interventions.

**Conclusion:**

Future research should prioritise adults living in low-income and middle-income countries, racialised individuals, as well as those with fewer educational opportunities. There is also a need to advance research in primary care settings, where longitudinal patient–provider relationships would facilitate the success of interventions.

## How this fits in

The literature on interventions targeting social isolation and loneliness among adults over 65 is robust. However, less attention has been paid to interventions specifically addressing social isolation among individuals between the ages of 18–64 within ambulatory healthcare settings. This systematic review identifies key elements of successful studies to inform future intervention design, which include delivery in a community health setting, the use of a group component, integrating CBT principles and psycho-educational components, and using technology to ensure that interventions are both long-lasting and flexible. Given the longitudinal relationship between patients and providers, and the opportunity for integrating wraparound and multidisciplinary care, primary care providers can play an instrumental role in implementing such interventions and addressing social isolation among their patients.

## Introduction

Social isolation is a target of intervention for public health researchers, given its associations with increased all-cause mortality,^1–6^ increased premature mortality,^7^ worse chronic disease management,^8,9^ and mental health concerns.^10^ For example, social isolation has been compared to smoking (15 cigarettes/day) and high levels of alcohol consumption (six drinks/day) as a predictor of mortality.^11^

Most of the research in social isolation interventions has focused on older populations. A systematic review of social isolation among older adults has shown that animal therapy and technology-based interventions had the largest effect on social isolation but found a low quality of evidence.^12^ While social isolation and loneliness is often thought of as a problem mainly affecting older populations, there is an increasing prevalence of social isolation among adults under 65 years of age.^13^ In a cross-country survey of adults in the US, UK, and Japan, it was found that the majority of people reporting loneliness were younger than 50.^14^ In adolescents and young adults, loneliness and social isolation have been associated with increased odds of asthma, migraine, arthritis, hypertension, depression and anxiety, alcohol use, and poor educational achievements.^15,16^ A recent call to action published in JAMA Psychiatry identified the need for clinical evaluations of interventions designed to enhance social connectedness.^17^ With the recent establishment of the World Health Organization Commission on Social Connection (2024–2026), addressing social isolation has risen to a global public health priority, prompting the scaling up of evidence-based solutions in countries of all incomes and across all age groups.^16^

Ambulatory care settings, particularly primary care settings, are strategically situated to identify and address social isolation in patients. Primary care settings are often the first point of contact between a patient and the healthcare system.^18–20^ Additionally, the provision of long-lasting, continuous care by primary care teams allows them to become a trusted source of health information for patients.^19^

To our knowledge, few studies have addressed social isolation interventions among the population under 65. Thus, in this systematic review, we aimed to identify interventions targeting social isolation in adults aged 18–64 and elucidate the role of ambulatory care settings, particularly primary care settings, in hosting and delivering such interventions. We further aim to distill key facets of effective interventions that address social isolation to make recommendations for future interventions.

## Method

We completed a systematic review of the literature to identify studies evaluating an intervention targeting social isolation delivered through ambulatory healthcare settings and describe key elements of effective interventions. This review was registered on PROSPERO (CRD42016049518).

### Search strategy

A search strategy was developed by an information specialist in consultation with the team, using a combination of subject headings and keywords adapted for each database, for the concepts of 'social isolation,' 'loneliness', AND 'ambulatory care'. Key terms were searched in the following databases: Ovid MEDLINE, Embase (OVID), The Cochrane Database of Systematic Reviews, Cochrane Central Register of Controlled Trials (Ovid), Scopus, CINAHL (EBSCOhost), and PsycINFO (Ovid). We retrieved articles published since database inception and September 27th, 2023 (the date of the final search). The search was limited to studies that included adults aged 18–64. Please see Supplementary Box 1 for the full search strategy.

### Study selection

The eligibility criteria were developed using PICO (Table 1). There were no restrictions on language, year, or study methodology.

**Table 1.** Inclusion and exclusion criteria

	Inclusion	Exclusion
Population	Adults 18 years and older.	Adults 65 years and older. To maintain focus on our target population, we excluded interventions that included individuals 18–64 but primarily targeted people 65 and over.
Intervention	Primarily focused on reducing social isolation and explicitly stated goal of programme. Intervention is implemented/delivered through ambulatory health care settings (for example, primary care clinic, walk-in clinic, out-patient specialist clinic, emergency department, mobile clinic, prison clinic). Patients are recruited through ambulatory healthcare setting, OR the intervention is delivered (all or in part) by staff of the ambulatory healthcare setting, OR the intervention is a partnership between an ambulatory healthcare setting and another agency.	Articles that did not have 'social isolation' or 'loneliness' as study outcomes were not accepted, even if the outcome was semantically related to social isolation, such as 'social inclusion', 'social integration', 'social connectedness', 'social support', 'social skills', and 'social functioning'. Inpatient or institutionalised settings were excluded.
Comparison	No intervention or standard of care.	
Outcome	Stated social isolation and/or loneliness as an outcome, measured via any previously validated instrument (for example, UCLA Loneliness Scale, De Jon Gierveld Loneliness Scale) or through a qualitative assessment.	
Types of study	Experimental, observational, mixed-methods, or qualitative studies.	Commentaries, opinion/editorials, reviews.
Other	There were no restrictions on language, year, or study methodology.	

### Screening

DistillerSR^21^ was used to manage articles during the screening process. In the first round of screening, title and abstracts were screened by two reviewers for relevance. In the second round of screening, the full text was reviewed by two reviewers to determine if it met the inclusion criteria. Any disagreements were settled by a third reviewer.

### Data extraction

The following information was extracted from each included article, using a standardised data extraction form (Supplementary Table 1): rationale, intervention, sampling technique, participant characteristics, outcomes related to social isolation or loneliness, results, limitations identified by the authors, and possible sources of bias.

### Quality appraisal

Each article was appraised by one to two reviewers to assess the risk of bias and methodological rigour. As this systematic review aimed to survey all available literature pertinent to our research question, we included all study designs. To ensure that quality appraisal was appropriate to the study methodology, we used four previously validated quality appraisal tools corresponding to the four distinct study designs in this review.

The Cochrane Risk of Bias tool was used to appraise experimental studies. The tool assesses seven domains of potential bias via a set of signalling questions, which is used by an algorithm to generate a risk of bias rating of 'Low', 'High', or 'Some concerns'.^22^

Observational studies were appraised using a nine-point rigour scale, adapted from an eight-point rigour assessment tool developed by The Evidence Project^22^ with scores ranging from 1 (high risk of bias) to 9 (low risk of bias), reflecting study quality.^23,24^

We used the Critical Appraisal Skills Programme (CASP) checklist for qualitative studies.^23^ The tool has ten questions, each focusing on a specific methodological aspect of the study. The relevant text from each study related to each CASP item was noted and rated by the reviewing author and cross-checked by a second author.

For mixed-methods research studies, we used the Mixed Methods Appraisal Tool (2018), which assessed five sources of bias for each type of study.^24^ Quantitative studies were assigned a score out of five, while mixed-methods studies were assigned a score out of 15. Quantitative studies with a score of four or five and mixed-methods studies with a score of 12 or above were considered to have a low risk of bias.

### Data synthesis

We conducted a narrative synthesis to identify common intervention types and key components of effective interventions. Quantitative and mixed-methods studies that showed a significant positive effect on social isolation and/or loneliness measures or qualitative studies reporting improvements in social isolation and/or loneliness were considered 'effective'. Those reporting mixed outcomes — for example, showing positive results over time but not directly due to the treatment — and those that failed to clearly report their findings were also noted.

## Results

### Study identification

**Figure 1.**
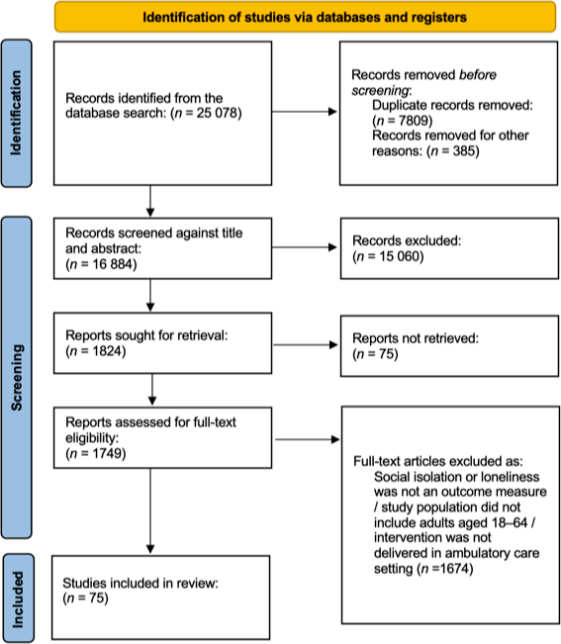
PRISMA study selection flow diagram

The literature search identified 16 884 citations after duplicates were removed. Title and abstracts were screened by two reviewers and 15 060 records were excluded. Full-text articles were screened by two reviewers, and 1674 articles were excluded. Following full-text screening, 75 articles met eligibility criteria and were included in the final review (Figure 1).

### Study characteristics

Study designs comprised 34 experimental studies,^25–58^ 20 observational studies,^59–78^ eight qualitative studies,^79–86^ and 13 mixed-methods studies.^87–99^ These articles were published from 1966–2023 inclusive.

Thirty-two studies were from the US,^25–27,29,31,34,38,39,42–44,47–49,52,55,59,64,66,67,69,73,75,78,81,86,88,93–95,99,100^ nine were from the UK,^36,51,57,70,79,83,89,90,98^ six were from Canada,^32,60,80,85,92,96^ six were from Australia,^40,58,62,63,65,97^ six were from the Netherlands,^28,33,37,56,77,87^ three were from Spain,^68,74,76^ one multi-centre study,^53^ and one each from China,^46^ the Czech Republic,^61^ Denmark,^91^ Iran,^45^ Ireland,^35^ Italy,^84^ Japan,^30^ Pakistan,^82^ Scotland,^72^ Singapore,^41^ Sweden,^71^ and Turkey.^54^

Most of the included studies had a specific population of focus. Thirty studies (*n* = 30, 40.0%) focused on individuals living with mental illnesses. These included individuals living with depression,^32,38,41,51,59,66,82,88^ social anxiety,^34,40,44,66,88^ psychotic disorder,^62,63^ schizophrenia,^61^ and other mental health conditions.^33,35,37,43,48,49,56,60,69,73,78,79,89,90,93,97^ Twenty-four studies (*n* = 24, 32.0%) focused on people living with chronic illnesses, such as HIV/AIDS,^27,31,52,80^ breast cancer,^29,30,42,55^ lung cancer,^46^ skin cancer,^87^ multiple sclerosis,^39^ type-1^91^ and type-2 diabetes,^81^ rheumatic diseases,^28^ stroke,^64^ aphasia,^36^ Parkinson’s disease,^53^ heart disease,^72,95^ liver disease,^76^ and multiple chronic conditions.^58,70,83,85^ Three studies (*n* = 3, 4.0%) focused on individuals living with chronic pain.^54,75,86^ Five studies (*n* = 5, 6.7%) focused on people living with a physical or intellectual disability.^25,26,50,67,98^ Two studies (*n* = 2, 2.7) focused on individuals experiencing homelessness, including one study on homeless youth and another on women living in shelters.^84,96^ Only one study focused on a Native American population (*n* = 1, 1.3%).^47^ Please see Supplementary Table 2 for the main characteristics of the included studies.

The following narrative synthesis categorises studies by common intervention components (group versus individual interventions, intervention setting, and use of technology) and type (psychological, psycho-educational, leisure and exercise, healthcare delivery, social care, befriending interventions). For each component and intervention type, we also assessed the effectiveness of the interventions, highlighting quantitative or mixed-method studies that reported a significant positive effect on social isolation and/or loneliness measures or qualitative studies that reported an improvement in social isolation or loneliness, as well as those reporting mixed and unclear results (Table 2).

**Table 2.** Synthesis of intervention effectiveness according to intervention component and type

Intervention component and type	No. of studies (%)	Quantitative studies with a significant positive effect or qualitative studies reporting improvement in social isolation or loneliness	Studies with no significant effect	Studies with mixed results	Studies with unclear results
Group vs individual					
*Group*	40 (53.3)	^25,27–30,40,44,45,51,56,61,63,65,68,69,74,77,78,82,84,85,89–92,97,98^ ^(*n* = 27, 67.5)^	^33,64,67,71,76^ ^(*n* = 5, 12.5)^	^31,39,59,80^ ^(*n* = 4, 10.0)^	^26,49,50,79^ ^(*n* = 4, 10.0)^
*Individual*	21 (28.0)	^38,42,43,46,47,53,62,66,70,73,75,83,95^ ^(*n* = 13, 61.9)^	^32,36,41,48,54,57,58^ ^(*n* = 7, 33.3)^	^35^ ^(*n* = 1, 4.8)^	
*Both*	14 (18.7)	^60,72,81,86,88,94,96^ ^(*n* = 7, 50.0)^	^37,52,55,87,93^ ^(*n* = 5, 35.7)^	^34^ ^(*n* = 1, 7.1)^	^99^ ^(*n* = 1, 7.1)^
Intervention setting					
Healthcare setting	23 (30.7)				
*Community or primary healthcare centres*	14 (18.7)	^29,38,56,60,61,63,70,74,81,83,88,89,92,97^ ^(*n* = 14, 100.0)^			
*Outpatient clinics*	9 (12.0)	^28,30,39,61,75,91^ ^(*n* = 6, 66.7)^	^55,64,76^ ^(*n* = 3, 33.3)^		
Community setting	18 (24.0)				
*Community organisations, service agencies, university psychology clinics*	16 (21.3)	^40,44,51,65,68,73,77,80,84,90,94,98^ ^(*n* = 12, 75.0)^	^35,71,93^ ^(*n* = 3, 18.8)^		^79^ ^(*n* = 1, 6.3)^
*Patient’s home*	2 (2.7)	^72^(*n* = 1, 50.0)	^57^(*n* = 1, 50.0)		
Virtual (patient’s home)	16 (21.3)	^25,27,42,43,85,95^ ^(*n* = 6, 37.5)^	^32,36,37,41,52,62,67,80^ ^(*n* = 8, 50.0)^	^26,31^ ^(*n* = 2, 12.5)^	
Multiple settings	5 (6.7)	^29,47^(*n* = 2, 40.0)	^48,58^(*n* = 2, 40.0)	^34^(*n* = 1, 20.0)	
Use of technology					
Used technology	21 (28.0)				
*Telephone or videoconferencing*	13 (17.3)	^25,27,29,42,43,85^ ^(*n* = 6, 46.2)^	^31,32,37,48,80^ ^(*n* = 5, 38.5)^		^26,67^ ^(*n* = 2, 15.4)^
*Smartphone application*	4 (5.3)	^43,62,94,95^(*n* = 4, 100.0)			
*Computer-based*	3 (4.0)	^84^(*n* = 1, 33.3)	^36,52^(*n* = 2, 66.7)		
*Internet forum*	1 (1.3)	^97^(*n* = 1, 100.0)			
Intervention type					
Psychological interventions	39 (52.0)				
*CBT*	13 (17.3)	^44,51,65,69,73,78,82,94,97^ ^(*n* = 9, 69.2)^	^55,76^ ^(*n* = 2, 15.4)^	^26,59^ ^(*n* = 2, 15.4)^	
*Counselling*	9 (12.0)	^25,38,42,84,98^(*n* = 5, 55.6)	^67,87^(*n* = 2, 22.2)		^49,50^ ^(*n* = 2, 22.2)^
*Peer support*	9 (12.0)	^88,91,92,95,96^ (*n* = 5, 55.5)	^32,41,93^(*n* = 3, 33.3)		^80^(*n* = 1, 11.1)
*Mindfulness training*	3 (4.0)	^43,44^(*n* = 2, 66.7)		^34^(*n* = 1, 33.3)	
*Didactic discussion*	2 (2.7)	^88^(*n* = 1, 50.0)	^93^(*n* = 1, 50.0)		
*Art therapy*	2 (2.7)	^89,90^(*n* = 2, 100.0)			
*Breath therapy*	1 (1.3)				^99^(*n* = 1, 100.0)
Psycho-educational interventions	39 (52.0)				
*Disease self-management and coping strategies*	30 (40.0)	^27,29,30,40,51,56,60,61,63,65,66,78,81,85,86,88,91,92,95–97^(*n* = 21, 70.00)	^33,64,71,87,93^(*n* = 5, 16.7)	^31,39,46,80^ ^(*n* = 4, 13.3)^	
*Enhancing self-efficacy via learning goal setting, problem-solving, and communication skills*	9 (12.0)	^28,45,63,77,92,94^(*n* = 6, 66.7)	^33,36^ ^(*n* = 2, 22.2)^	^39^ ^(*n* = 1, 11.1)^	
Leisure & exercise interventions	4 (5.3)				
*Exercise-based intervention*	3 (4.0)	^47,65^(*n* = 2, 66.7)	^58^(*n* = 1, 33.3)		
*Leisure*	1 (1.3)	^68^(*n* = 1, 100.0)			
Healthcare delivery intervention	6 (8.0)	^53,70,75^(*n* = 3, 50.0)	^48,54^(*n* = 2, 33.3)		^79^
Social care intervention	3 (4.0)	^60,74,83^(*n* = 3, 100.0)			
Befriending intervention	3 (4.0)	^72^(*n* = 1, 33.3)	^57^(*n* = 1, 33.3)	^35^(*n* = 1, 33.3)	

NOTE: multiple intervention components and types were considered in some studies.

CBT = Cognitive behavioural therapy;

### Intervention effectiveness according to intervention components

#### Group vs. individual interventions

A key finding was the effectiveness of group interventions, with over half (*n* =40, 53.3%) incorporating a group component,^25–31,33,39,40,44,45,49–51,56,59,61,63–65,67–69,71,74,76–80,82,84,85,89–92,97,98^ and the majority (*n* = 27) yielding significant improvements in reducing loneliness and/or social isolation.^25,27–30,40,44,45,51,56,61,63,65,68,69,74,77,78,82,84,85,89–92,97,98^ Individual interventions (*n* = 21, 28.0%),^32,35,36,38,41–43,46–48,53,54,57,58,62,66,70,73,75,83,95^ were also notable, with 13 demonstrating effectiveness.^38,42,43,46,47,53,62,66,70,73,75,83,95^ Combined group and individual interventions (*n* = 14, 18.7%),^34,37,52,55,60,72,81,86–88,93,94,96,99^ showed a split in effectiveness, with half reporting significant results.^60,72,81,86,88,94,96^

#### Intervention setting

Most studies took place in healthcare settings (*n* = 23, 30.7%), with those in community health or primary care centres showing the most effectiveness.^38,56,60,61,63,70,74,81,83,86,88,89,92,97^ Many effective interventions aimed to foster a network between primary care and specialists, as well as other community agencies. For example, one study aimed to strengthen the connection between primary care physicians and psychiatrists to prevent social isolation among individuals diagnosed with a schizophrenia-spectrum disorder.^61^

Nine studies (*n* = 9, 12.0%) took place in outpatient clinics,^28,30,39,55,61,64,75,76,91^ of which six improved loneliness and/or social isolation outcomes.^28,30,39,61,75,91^

Interventions set in community settings (*n* = 18, 24.0%), including at a university psychology clinic,^40,44,73^ a daily care centre,^68^ a senior service agency,^77^ an arts organisation,^90^ a learning disability trust,^98^ and other unspecified community settings,^51,65,80,84,94^ showed considerable effectiveness.

Two studies took place in the patient’s home and featured a volunteer befriending and support service.^57,72^ Peardon *et al* (2010) provided direct social support and regular open meetings between patients and caregivers.^72^ Patient feedback from open-ended questionnaires indicated a positive impact on social isolation.^72^ Walshe *et al* (2016) provided a befriending intervention to patients eligible for end-of-life care.^57^ The authors observed a slower decline in loneliness within the intervention group than the usual care group, although the difference was not statistically significant.^57^

Sixteen studies (*n* = 16, 21.3%) featured an intervention delivered virtually from the patient’s home, of which only six were effective.^25,27,42,43,85,95^ Five studies were set across multiple settings (*n* = 5, 6.7%).^29,34,47,48,58^ Two of these were effective, including one study that combined individual telephone and group support and education in a healthcare setting,^29^ and another study that took place at a rural Chapter House, an urban church, and an urban Native American-serving federally qualified health centre.^29,47^ Two studies showed no significant effect, including one randomised control trial that delivered caring text messages to healthcare providers, staff, and patients,^48,58^ and another that compared gym-based and home-based exercise with telephone support.^58^ One study targeting adults with generalised social anxiety disorder provided meditation and Hatha yoga at a public health centre, with virtual at-home options, and reported mixed results.^34^

#### Use of technology

Technology played a role in nearly a third of the interventions (*n* = 21, 28.0%).^25–27,29,31,32,37,42,43,48,50,52,58,67,80,83–85,94,95,97^ Thirteen studies used a telephone or videoconferencing-based approach,^25–27,29,31,32,37,42,43,48,67,80,85^ of which six were effective in reducing loneliness and/or social isolation measures.^25,27,29,42,43,85^ All four studies that used a smartphone application were effective. These studies used smartphones to deliver videos modelling psychology concepts,^62^ mindfulness training,^43^ psychosocial telehealth sessions,^95^ and daily psycho-educational messages to participants.^94^ Three studies used a computer-based approach.^36^ Of these, only one qualitative study featuring open-ended group meetings with women living in shelters was effective. One study used an internet forum to facilitate communication between volunteers and individuals diagnosed with a gambling disorder and reported significant improvements in loneliness from baseline to six months (*P* =0.003).^97^

### Intervention effectiveness according to intervention type

#### Psychological interventions

Many studies (*n* = 39, 52.0%) featured a psychological intervention that addressed the association between mental health and social isolation. Those using integrated cognitive-behavioural therapy (CBT) approaches (*n* = 13, 17.3%) to challenge negative thoughts and reduce psychological distress, were most effective.^44,51,65,69,73,78,82,94,97^ Other psychological interventions, including counselling (*n* = 9, 12.0%)^25^ and peer support (*n* = 9, 12.0%)^32,41,88,91–93,95,96^ demonstrated over fifty per cent effectiveness. Peer support was effective in fostering social support for people living with type-1 diabetes,^91^ HIV/AIDS,^88^ homeless youth,^96^ and expectant mothers of babies diagnosed with congenital heart disease.^95^

Fewer studies examined the effectiveness of other psychological interventions such as mindfulness training (*n* = 3, 4.0%),^34,43,44^ didactic discussions (*n* = 2, 2.7%),^88,93^ art therapy (*n* = 2, 2.7%),^89,90^ and breath therapy (*n* = 1, 1.3%).^99^ Both art therapy interventions were effective in reducing feelings of social isolation among participants.^89,90^

#### Psycho-educational interventions

Over half the studies (*n* = 39, 52.0%) featured a psycho-educational intervention. Of these, 40% (*n* = 30) featured self-management and coping strategies, for people living with a chronic disease,^27,29–31,39,46,64,65,71,80,81,85–88,91,92,95^ mental illness,^33,40,56,60–62,66,78,93,97^ severe disability,^50^ and those facing homelessness.^96^ Most of these studies (*n* = 21) were effective.^27,29,30,40,51,56,60,61,63,65,66,78,81,85,86,88,91,92,95–97^ Nine studies (*n* = 9, 12.0%) focused on enhancing self-efficacy through strategies like goal setting, problem-solving, and communication skill development,^28,33,36,39,45,63,77,92,94^ achieving effectiveness in six of the studies.^28,45,63,77,92,94^

#### Leisure and exercise interventions

Leisure and exercise-based interventions, although fewer (*n* = 4), were largely effective (*n* = 3).^47,58,65^ Bea *et al* (2023) examined the feasibility of a culturally tailored exercise programme on cancer-risk biomarkers and quality of life among Native American cancer survivors and reported improvements in isolation subscale scores across cohorts (*P*<0.05).^47^ Deans *et al* (2021) incorporated a one-hour group physical activity session, noting a significant reduction in average UCLA-Loneliness scores between the programme’s start and the post-programme assessment (*P*<0.005).^65^ Garcia *et al* (2003) featured leisure-based weekly activity workshops involving gymnastics, computer science, and arts, and found significant decreases in loneliness scores (*P*<0.01).^68^

#### Healthcare delivery interventions

Six studies (*n* = 6, 8.0%) delivered specialised healthcare services to patients to improve social isolation and loneliness. Interventions included home-based health services for individuals living with HIV/AIDS,^52^ mental health care,^37^ ultrasound therapy for cervical myofascial pain syndrome,^54^ acupuncture,^70,75^ and carbidopa medication for individuals with Parkinson’s disease.^53^ Acupuncture services delivered through primary care was shown to effectively reduce social isolation and loneliness among primary living with chronic pain and chronic diseases.^53,70,75^

#### Social care interventions

All three studies (*n* = 3, 4.0%) that integrated action on social determinants of health were effective. Wildman *et al* (2019) investigated the experiences of patients with long-term conditions utilising a social prescribing service within primary care in a socio-economically deprived region of Northeast England. The authors found decreased levels of social isolation per self-report.^83^ Petryshen *et al* (2001) implemented a multi-level intervention featuring an environmental change initiative to support a community mental health programme.^60^ Participants reported statistically significant lower levels of loneliness at one-year follow-up.^60^ Coll-Planas *et al* (2015) implemented a coordinated action strategy that involved building a network between primary healthcare centres, senior centres, and other community assets where older people could participate in activities.^74^ The long-term impact evaluation showed that loneliness had reduced significantly (*P*<0.001).^74^

#### Befriending intervention

Befriending interventions (*n* = 3, 4.0%) for patients in their last year of life,^35,57,72^ those living with chronic heart failure,^72^ and individuals with a serious mental illness^35^ had mixed results. Only one observational study by Peardon *et al* (2010) demonstrated a significant positive effect.^72^

### Quality appraisal

We assigned each study an overall quality rating of 'low risk', 'high risk', or 'some concerns' of bias based on the results of the quality assessment. Fifteen studies had low risk of bias (20%), 12 had a high risk of bias (16%), and 48 studies had some concerns of bias (64%). For quality appraisal of literature and risk of bias findings see Supplementary Table 3.

## Discussion

### Summary

Our findings indicate that group interventions, delivered in primary care and community health centres, are effective in reducing social isolation and loneliness. Digital technologies, particularly telephone or videoconferencing and smartphone applications, enhance the flexibility and efficacy of interventions. Effective interventions also focused on addressing mental health through CBT-based psychological interventions. Peer support and counselling also played a key role in enhancing social support and social integration. Psycho-educational interventions focusing on self-management for chronic conditions also showed effectiveness in social isolation and loneliness outcome measures.

### Strengths and limitations

This review fills a gap in the existing literature by examining social isolation interventions targeting community-dwelling adults below the age of 65 in ambulatory healthcare settings. Our comprehensive search strategy and inclusion criteria ensured that we captured extensive literature pertinent to the subject, covering a wide range of study designs. To ensure that quality appraisal was appropriate to the study methodology, we used four previously validated quality appraisal tools appropriate for the four distinct study designs in this review.

Limitations include the poor quality of some studies, and the variability in measurement tools, notably self-report measures prone to bias, which can affect the assessment of intervention effectiveness. Furthermore, while our review included a variety of sub-populations, ages 18–64, the focus on specific groups in most interventions may limit the generalisability of our findings.

### Comparison with existing literature

Most research in social isolation interventions has focused on older populations. Many reviews have focused on the effectiveness of digital technologies,^101–103^ especially considering the periods of mandated social isolation during the COVID-19 pandemic.^104^ A recent meta-review of social prescription interventions for older adults found that group interventions, particularly those incorporating peer support, were effective in reducing social isolation and loneliness among migrants and individuals living with a disability.^105^

Reviews on social isolation interventions for adults under 65 are limited. Similar to our findings, one systematic review found that technology and support groups are important in reducing loneliness among non-elderly adults.^106^ Masi *et al* (2011) found that group-based interventions bolster social contact and support, while technology-based programmes address maladaptive therapy and increase social support.^107^ Osborn *et al* (2019) found interventions in institutional environments like educational and healthcare settings particularly effective for young people.^108^

Primary care settings are amenable to leveraging social isolation as a target of intervention, owing to their unique niche as the ‘patient medical home’ and the potential for integrating multidisciplinary care. Few reviews have focused on interventions implemented specifically in primary care settings, and none identified have been on adults under 65 years old.^105,109^ A recent scoping review by Galvez-Hernandez *et al* (2022) showed that despite the growing collaboration between primary care and non-healthcare sectors, more effort should be made to tailor interventions to older adults’ social needs and to design long-lasting interventions that foster meaningful social networks.^18^

The COVID-19 pandemic has exposed larger segments of the population to the risk of social isolation and loneliness, underscoring the need for interventions among the wider public. A systematic review of interventions compatible with social distancing measures found that effective interventions integrated psychological therapies, social skill building, and social facilitation.^110^ However, few interventions improved social isolation. Understanding varied experiences of loneliness and isolation during the pandemic is needed.^110^

### Implications for research and practice

We identified few studies aimed at populations made vulnerable by social and economic policies. This includes those living in low- and middle-income countries, where the prevalence of social isolation is on par with or higher than in high-income countries.^16^Additionally, individuals from racialised communities and those with fewer educational opportunities face a greater risk of social isolation and loneliness.^111–114^ Future research should focus on these groups with culturally sensitive and age-specific interventions to meet their unique needs.

Further research is also needed on the role of primary care in addressing social isolation. Integrating social isolation interventions in these settings enables practitioners to simultaneously address patients’ immediate health concerns and underlying social determinants, such as homelessness.^18^ As a space for social support and community resources, primary care can play a larger role in effectively identifying and mitigating social isolation.
